# The Influence of Diabetes Mellitus on the Outcome of Superficial Femoral Artery Recanalization is Debatable

**Published:** 2020-02-20

**Authors:** L Rizzo, A D’Andrea, N Stella, P Orlando, M Taurino

**Affiliations:** 1 Vascular Surgery Unit, Sant’Andrea Hospital, University of Rome “La Sapienza”, Italy

**Keywords:** peripheral arterial disease, diabetes mellitus, critical limb ischemia, endovascular procedure

## Abstract

Notwithstanding technological improvements in endovascular devices treatment of steno-obstructive lesions of the superficial femoral artery (SFA) remains a challenge for today’s vascular surgeon. Current opinion dictates that the diabetic population may have worse outcome after revascularization of the lower extremities. Herein we examine the effects of endovascular treatment on steno-obstructive lesions of the SFA in diabetic and non-diabetic patients.

**Methods:**

A retrospective analysis was carried out on 110 patients who had undergone endovascular treatment of the SFA from 2010 to 2017 comparing outcomes in diabetic (DM) *vs* non-diabetic patients (nDM).

**Results:**

56 (50.9%) of the patients were diabetic and 54 were non-diabetic (49.1%). 52.7% (62.7% DM vs 35.2% nDM, p = 0.0003) were patients with critical limb ischemia. SFA occlusion was present in 65.5% (60.7% DM vs 70.4% nDM, p = 0.29) of all patients. All had undergone PTA of the SFA and 40.9% had received adjunctive stenting (44.6% DM vs 37.0% nDM, p = 0.41). A multilevel treatment was executed in 39.1% (51.8% DM vs 25.9% nDM) of the cases whereas an infra-popliteal procedure was associated in 27.3% (37.5% DM vs 16.7% nDM). In both groups the presence of diabetes was significantly associated (p = 0.005 e p = 0.014, respectively). Reintervention rate was 22.7%; 13 in the diabetic group (23.2%) and 12 in the non-diabetic group (22.2%). Of those who had had reintervention (p = 0.77); 9 patients (8.2%) had undergone an open surgical operation, 6 of whom had diabetes (p = 0.32). 5 patients (4.5%) had had major amputation, 4 of whom were diabetic (p = 0.20). Curves assessing freedom from target lesion restenosis were substantially overlapping between the two groups.

**Conclusion:**

No statistical associations between diabetes and reintervention or amputation rates were found. Indication to treat the SFA were not influenced by the presence of diabetes but further investigation is required to verify our hypothesis.

## I. INTRODUCTION

The superficial femoral artery (SFA) still today remains a great challenge for the Vascular Surgeon. Over the last few years’ innovations and technological developments in the endovascular field such as drug coated or atherectomy devices^1^, interwoven stents, intravascular lithotripsy, etc., have certainly increased. Despite this, long-term outcomes are not fully satisfactory. A recent series shows a patency rate of 51% at 3 years after percutaneous intraluminal angioplasty (PTA) and provisional stenting of the SFA.^2^ The presence of complex lesions (TASC II D) negatively affects the results with primary patency rates of 60.1% at 1 year, 50.6% at 3 years and limb salvage rates of 63.7% at 4 years.^3^ Even the use of drug coated devices does not seem to improve these data; a single-centre study shows patency rates of 56% and 39% at 1 and 2 years, respectively.^4^ The reasons for this poor success have been ascribed to the anatomic peculiarities of the SFA which is prone to deformations such as kinking, twisting and external compression during the flexion-extension movements of the knee. These forces may be responsible for restenosis following endovascular procedure.^5^–^6^ Moreover, the highly calcified nature of the atherosclerosis plaques in the femoropopliteal segment is well known and seems to be correlated to a young-age medial tunica calcinosis.^7^ Several studies show that patency after endovascular intervention is linked to degree of calcification.^8^–^9^

Diabetic patients are well known for the severity and precocity of peripheral arterial disease (PAD) with a major risk of amputation. This is due to significant changes in the composition and function of the arterial wall caused by a dysmetabolic state^10^–^11^ and also for the peripheral neuropathy and local susceptibility to infections. In fact, amongst diabetic patients PAD has unique features such as early age onset, simultaneous arteriopathy in other segments, aggressive progression, high incidence of calcified lesions, distal and microcirculatory involvement and reduced collateral reserve.^12^–^13^ This becomes even more relevant if we consider that diabetes mellitus not only represents a highly invalidating disorder with terrible complications, but also the fact that its worldwide diffusion continues to increase with a global prevalence expected to rise to 552 million by 2030. 14 Endovascular treatment has a growing application among diabetics who are often not eligible for open surgery due to the absence of distal target vessels for a peripheral bypass and the coexistence of several comorbidities and poor general conditions.^15^–^16^ However, diabetic patients show diminished primary patency rates after endovascular revascularization of the lower extremities.^17^ These data are even worse in cases of poor perioperative glycaemic control.^18^ In addition, a failed endovascular treatment could have a negative impact on the success of a potential subsequent bypass^19^ given the frequent lack of run-off vessels among diabetic patients. For these reasons an endovascular approach to this challenging segment of diabetic patients could be presumed to have poor success but in reality the data in the literature is not univocal.

The aim of this study is to examine a population of patients with PAD who had undergone endovascular treatment of SFA and compare the outcomes between the diabetic (DM) and non-diabetic (nDM) patients.

## II. METHODS

A retrospective analysis on 110 patients with PAD who had undergone endovascular treatment on SFA was conducted. The study included patients admitted to a tertiary referral academic hospital from January 2010 and December 2017. Patients who had experienced 1) claudication with mean walking distance < 200 meters not responsive to medical therapy and daily exercise, 2) resting pain and 3) ulcer or necrosis of lower extremities were all included. All patients presented with atherosclerosis lesions of the SFA, with or without the involvement of other arteries of the same limb.

The decision to treat patients with an endovascular procedure of the SFA as first line was based on clinical examination and lesion morphology assay. Before surgery, diagnostic evaluations comprehensive of lab tests, two view chest radiographs, cardiologic and anesthesiological assessments were carried out. A clinical and hemodynamic examination of the lower limbs comprehensive of the Ankle-Brachial Index (ABI), was also made. The presence of values < 0.40 was established as indication of severe peripheral vasculopathy. A measure of pre- and post-procedural Transcutaneous Oximetry (TcPO2) was realized on most patients with trophic lesions.

A preoperative Computed Tomography Angiography (CTA) was given to most patients to investigate arterial lesions: differentiation between stenosis and obstruction, length of lesions, presence of highly calcified plaques and the conditions of run-in and run-off vessels were all examined. These aspects played an essential role in indication to treat. In other cases, a lower extremities Arteriography made at the same time as treatment.

The endovascular procedures were performed in the Operatory Room or in the Interventional Radiology Room. Patients with chronic kidney disease underwent pre- and post-operative hydration. Local anesthesia with sedation was administered to almost all patients. In a minority of cases general or subarachnoid anesthesia was administered. Access to the SFA was most frequently obtained with a retrograde ultrasound-guided puncture of the contralateral common femoral artery (CFA) and crossover approach. In selected cases an anterograde puncture of the CFA or a left brachial artery access was achieved. In only one case a retrograde puncture of the anterior tibial artery was associated with traditional access. In most cases a PTA of the atherosclerosis lesion was realized after injection of iodinated contrast media. Different devices were chosen, depending on the attending physician’s preference and of an appropriate size and length according to the anatomy of the lesions (range 4–7 mm and 20–220 mm, respectively). Duration of dilation varied between 1 and 2 minutes.

Stent deployment was more often utilized as “rescue therapy” in cases of unsatisfactory PTA results (residual stenosis > 30% or re-stenosis) or of flow limiting flap / dissection. Primary stent placement was preferred in the presence of long chronic total occlusion of the SFA. In other cases, the choice of device fell to drug coated balloons and/or atherectomy in the presence of highly calcified lesions when stent positioning was not recommended or not considered by preference of the physician.

In some cases, treatment of the SFA was associated with an open-surgery or endovascular treatment on other arteries of the same limb consisting of endarterectomy of the CFA +/− profundoplasty, iliac PTA +/− stenting, or PTA of the popliteal or tibial arteries. Adjunct procedures were meant to improve the SFA inflow or outflow and were considered based upon the presence of trophic lesions, patient’s general conditions and lifestyle.

In this study patients with acute-on-chronic ischemia caused by plaque thrombosis were also included. In such conditions loco-regional fibrinolysis, aspiration thrombectomy and/or sodium heparin injection were initially performed.

During the post-operative period antiplatelet therapy and, in cases of stenting, double antiplatelet drugs were administered for 6 months.

Diabetes mellitus was defined based upon blood glucose level measurement and confirmed by antidiabetic drug consumption (Oral Hypoglycemic Agents and/or insulin). In 8 patients presence of diabetes mellitus was discovered during hospitalization for treatment of PAD. Presence of arterial hypertension was assessed by arterial pressure measurement and antihypertensive drugs. Dyslipidemia was defined on the basis of lab tests and/or antilipemic drug consumption. Chronic kidney disease was defined by presence of creatinine blood level > 1.5 mg/dL and/or dialyze. Coronaropathy was considered in relation to anamnestic history of coronary artery revascularization.

Before hospital discharge, peripheral pulse examination, ABI measurement, pain-free walking distance and resting pain evaluation were all evaluated. Trophic lesions were assessed in the long term through outpatient medications. Our study considered post-operative ABI increasing by more than 0.10 as significant. Patients had undergone duplex follow up at 1–3–6–12 months after procedure. Target lesion patency, measure of restenosis or residual stenosis and downstream flow were evaluated. Hemodynamically significant stenosis was defined by a PSV ratio > 2.5 between upstream and downstream PSV in relation to target lesion.

Medium follow-up was 17.9 months (range 0–96.4). Procedure related mortality was 3.6% (3.6% DM vs 3.7% nDM)

Primary endpoints were hemodynamic improvement, the presence of hemodynamically significant restenosis or re-occlusion of target lesion, endovascular or open-surgery reintervention in the femoro-popliteal segment and limb salvage. Exclusion criteria were procedure failure, previous endovascular or open-surgery procedures on the SFA and cases of intrastent restenosis treatment.

All data are reported as numeric values or percentages. Chi-square analysis and the Fisher test were used to evaluate differences between diabetic and non-diabetics patients. All values of p < .05 were considered statistically significant. The analysis of freedom from hemodynamical restenosis of target lesions was performed using Kaplan Meier life tables.

## III. RESULTS

Total number of patients in the study were 110 of which 56 (50.9%) were diabetic and 54 (49.1%) non-diabetic.

### Demographics

**Figure 1:**
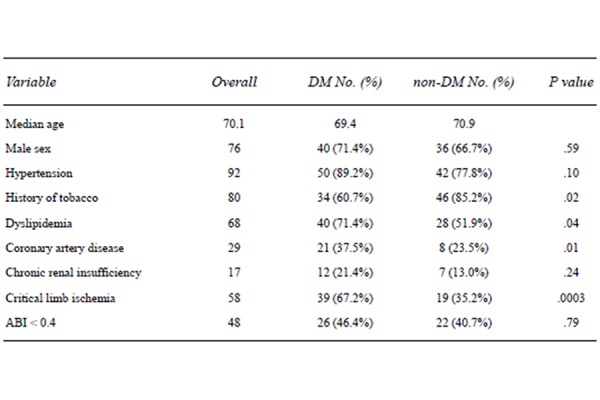
Demographics and clinical data.

Average age was 69.4 years in the DM group vs 70.9 years in the nDM group. All data for DM, nDM and total number of patients are given in Figure 1. A statistically significant association was found between diabetes and the presence of dyslipidemia (71.4% DM vs 51.9% nDM, p = 0.035) and coronaropathy (37.5% DM vs 23.5% nDM, p = 0.014). Surprisingly, a history of smoking was more frequent in the nDM group, with a significant relationship (60.7% DM vs 85.2% nDM, p = 0.024). 52 patients suffered from claudication and 58 from critical limb ischemia (CLI). As expected, a statistically significant relationship was found between diabetes and CLI (67.2% DM vs 35.2% nDM, p = 0.0003). ABI showed values consistent with severe PAD (<0.40) in 46.4% of DM vs 40.7% of nDM, although without a strong relationship (p = 0.79).

### Lesion characteristics

In 72 patients (65.5%) preoperative imaging showed an SFA occlusion, particularly in 60.7% of the DM and 70.4% of the nDM (p = 0.29) group. The mean length of occlusion was 9.4 cm (range 1–30 cm); 8.6 cm (range 1–25 cm) in the DM group and 10.2 cm (range 2–30 cm) in the nDM group. The mean lesion length in general (stenotic and obstructive together) was 10.9 cm (range 1–30 cm); 10.6 cm (range 1–30 cm) in the DM group and 11.2 cm (range 2–30 cm) in the nDM group.

**Figure 2:**
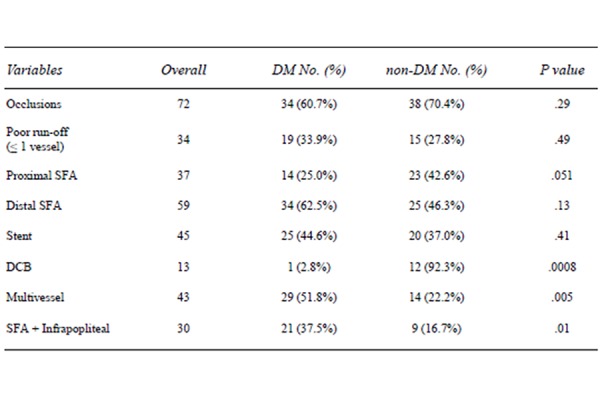
Characteristics of lesions and treatment

In 37 patients (33.6%) the proximal third of SFA was involved (25.0% DM vs 42.6% nDM) with a near-significant trend (p = 0.051). Out of this group patients with unique short lesions at the origin of the SFA with femoral bifurcation involvement were excluded; in those patients a femoral endarterectomy was offered. Conversely, in 59 patients (53.6%) the level of lesion was the distal third of the SFA (62.5% DM vs 46.3% nDM, p = 0.13). Patients with continual involvement of the supragenicular popliteal artery were included with the exception of those with isolate popliteal lesions. (Figure 2)

Preoperative tibial run-off defined as number of patent tibial vessels to the foot was assessed. 23 patients had only one patent vessel and 11 patients had an absence of run-off tibial arteries accounting for 34 patients (30.9%) considered to have poor run-off. 33.9% of the DM group and 27.8% of the nDM group had only ≤ 1 run-off vessels (p = 0.49).

### Treatment

All patients received PTA of SFA. In 13 patients a drug coated balloon was employed. Interestingly, only one of these was diabetic with a statistically significant relationship (p = 0.0008). In 9 cases the artery was preoperatively occluded. In 6 patients an atherectomy was also carried out, 2 in the diabetic patients. In 45 patients (40.9%) stent placing was associated to PTA. Of these, 25 patients were diabetic and 20 non-diabetic (44.6% DM vs 37.0% nDM, p = 0.41). In 36 cases there was a preoperative obstruction of the SFA. Only nitinol self-expandable stents were positioned, almost all uncovered. In 3 cases (n. 2 DM) a covered stent was released. The mean diameter of stent was 5.9 mm (range 5–7 mm). In most cases only one device was placed/inserted. In 7 patients 2 stents were released (n. 4 DM) and in 2 patients 3 stents (n. 1 DM) were released. No drug eluting stents were placed.

In 43 patients (39.1%) a multivessel treatment was performed (51.8% DM vs 25.9% nDM) with this association being statistically significant (p = 0.005). 13 patients were offered an adjunctive iliac or femoral procedure (8 DM vs 5 nDM), otherwise 30 patients received an adjunctive infragenicular popliteal and/or tibial procedure (21 DM vs 9 nDM) with statistical significance (p = 0.014).

### Follow up

**Figure 3:**
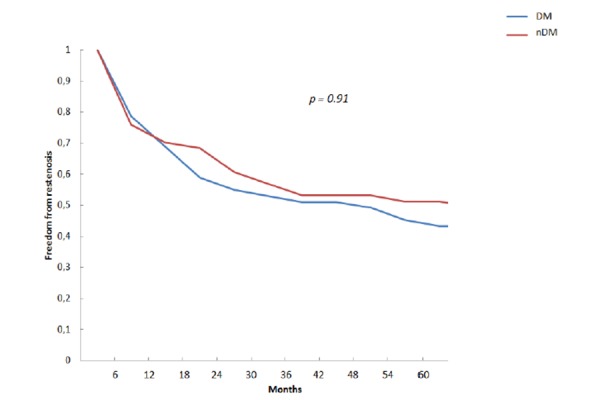
Kaplan-Meier curves for freedom from restenosis at 5 years for diabetics (DM) and non-diabetics (nDM)

Kaplan-Meier curves for freedom from hemodynamical restenosis of target lesions for diabetics and non-diabetics were formulated. (Figure 3). The curves were substantially overlapping between the two groups; a log rank test showed absence of a significant relationship (p = 0.91). One month after procedure, freedom from restenosis was major in the DM group (91.7% DM vs 83.7% nDM, p = 0.23). After 6 months this trend inverted (72.9% DM vs 75.5% nDM, p = 0.77). Even in the long term, freedom from restenosis rates were substantially similar between the two groups with a slightly worse trend among diabetics: 62.5% DM vs 73.5% nDM (p = 0.25), 54.2% DM vs 57.1% nDM (p = 0.77), 45.8% DM vs 53.1% nDM (p = 0.41) at 1, 2 and 5 years, respectively. An increase of ABI > 0.10 on the first postoperative day was present in 83.6% (80.4% DM vs 87.0% nDM, p = 0.34). In 36 patients values of TcPO2 increased from 12.3 +/− 7.1 to 39.3 +/− 11.6.

During the entire follow up rates of total reinterventions on target lesions, open surgical reinterventions and lower limb amputations were 22.7% (23.2% DM vs 22.2% nDM, P = 0.77), 8.2% (10.7% DM vs 5.6% nDM, P = 0.49) e 4.5% (7.1% DM vs 1.9% nDM, P = 0.36), respectively and no statistically significant differences were found. In cases of endovascular reintervention, a re-PTA, with or without stenting, was executed. In the presence of intrastent thrombosis this procedure was preceded by loco-regional fibrinolysis. In cases of open surgical conversion, a femoro-popliteal bypass, above or below the knee, was realized in almost all of the patients. In only one case a profundoplasty with patch enlargement was done.

**Figure 4:**
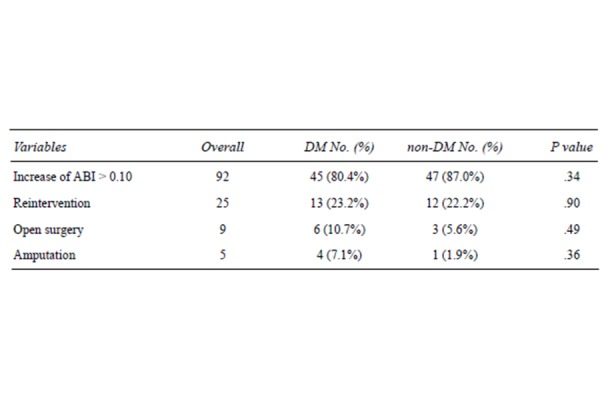
Hemodynamical improvement, reintervention and amputation rates during the entire follow up

Also amongst the claudicants any statistical association was found between diabetes and reintervention rate or open surgical operation (p = 0.96 and 0.31, respectively). Major amputation rate was 0%. (Figure 4).

## IV. DISCUSSION

In the present study no statistically significant differences in the outcome of SFA revascularization between the diabetic and non-diabetic patients were found although the trend was slightly worse in the DM group. A supposition of the main reason for this could be in the combination of patient characteristics and lesions for which endovascular treatment was initially recommended. Endovascular treatment was preferred in cases of anatomic lesions which, from our point of view, could have benefited. The same procedure was also performed in patients considered unfit for surgery. For patients with more complex lesions a femoral-popliteal bypass was chosen as first choice however these latter are not considered in the current study.

Darling et al. made a comparison between an “endovascular first” vs “bypass first” strategy on a femoral popliteal segment in patients with insulin dependent diabetes with CLI; “bypass first” patients had significantly lower unadjusted 6-month rates of incomplete wound healing (49% vs 57%) and 5-year rates of restenosis (53% vs 72%) and reintervention (47% vs 58%; all P < .05)., with perioperative complications not differing.^20^

In our study, rates of total reinterventions (22.7%), open surgical reinterventions (8.2%) and major amputations (4.5%) are comparable to the data in the literature. Amputation rates of 5% are reported by other authors^21^ and another study shows limb-salvage rates of 90% at 24 months.^22^ Shammas et al. reported a reintervention rate of 17% at 2 months.^23^ Data concerning freedom from restenosis are also comparable to the literature, with rates of 62–79% and 37–55% at 1 and 5 years, respectively.^24^

In a study of 1075 endovascular procedures on SFA with mean follow up time of 34 months, Abularrage et al. found rates of 5-year primary patency (PP) and limb salvage significantly worse amongst diabetics (37% vs 46% P = 0.009 and 84% vs 93% P = 0.0001, respectively) despite the fact that the assisted patency (PA) was did not differ. The authors concluded that diabetes is an independent predictor of decreased long-term PP and that strict surveillance could improve the PA.^25^ A previous study of the same group with mean follow up time of 24 months did not show significant differences between the two populations.^26^ The explanation of the authors for this is the reduced period of post-operative surveillance. In our study the curves for freedom from hemodynamical restenosis substantially overlap between the two groups, with even better results amongst diabetics during the first 6 months. These data support the hypothesis that diabetes could have a negative impact, especially on long-term outcomes of these procedures, but further studies are needed.

Shammas et al., in a similar study, found a significant relationship between diabetes and amputation at 12 months (HR 5.02, 95% CI 1.44 to 17.56, P = 0.011) with increased risk among patients with CLI. However, no differences of reintervention rates were found.^23^

Several studies on the efficacy of SFA endovascular treatment consider diabetes as predictive of patency failure.^22^, ^27^–^29^

Bodewes et al. reported that diabetes was a predictive factor of 30-day unplanned hospital readmission after infrainguinal endovascular intervention due to non-healing wounds, infections or recurrence of symptoms. The authors suggested strict surveillance and prophylactic measures among categories at risk.^29^ Conversely, Xiao et al. reported the complete absence of significant differences between diabetics and non-diabetics in terms of PP (73.2% DM vs 80.9% nDM, 58.6% DM vs 52.8% nDM, at 1 and 3 years respectively) and limb salvage (81.9% DM vs 83.1% nDM at 3 years), showing a similar efficacy of endovascular treatment in these two groups.^31^

In a study by Bakken et al., patients who had undergone endovascular procedure on SFA were divided into non-diabetics, non-insulin-dependent diabetics and insulin-dependent diabetics and were then separately considered in two categories: claudicants and CLI-affected. Among claudicants no significant difference in PP existed between the three groups, even with increased rates of PP (72% vs 51% at 5 years) and freedom from restenosis (68% vs 57% at 3 years) between the non-insulin-dependent diabetics and the non-diabetics. Among patients with CLI, no significant differences were noted between the three groups in PP, PA and freedom from restenosis, with primary patency rates found to be even higher among insulin-dependent diabetics compared to non-diabetics (57% vs 30% at 3 years).^24^ A review of Ihnat et al. on endovascular treatment of the lower limb amongst diabetics concluded that patients who had undergo intervention for SFA lesions are a very heterogeneous group and further studies are required to understand the impact of diabetes on outcomes. The role of diabetes itself in patency and restenosis rates is probably less important than other factors such as TASC classification and tibial run-off.^32^

In our experience diabetes was significantly associated with dyslipidemia and coronaropathy and inversely correlated with smoking history. This latter finding could be explained by the role of smoking prevention and discouragement campaigns which are displayed in our diabetic foot ambulatory Care clinic. On the other hand, it is likely that patients who were both diabetics and smokers were more frequently judged not eligible for endovascular treatment or underwent direct amputation due to the major complexity of arterial lesions and the worsening clinical conditions of limbs.

In this study, as in others, an association between diabetes and CLI was found. In fact, in several articles the presence of CLI was a negative predictor of failure of SFA endovascular intervention and amputation.^21^,^25^ Shammas et al. found an association between diabetes and limb loss only among patients with CLI.^23^ In our study attention was primarily focused on the role of diabetes itself regardless of clinical conditions of the lower limb.

In the same way attention was turned to the presence of diabetes or not without previously considering the nature of arterial lesions. This may explain the absence of association of diabetes with length of lesions or presence of occlusions. More complex lesions did not usually receive endovascular procedures. In fact, there were only 9 cases of obstructive lesions of the SFA longer than 20 cm. In addition, other studies also concur with the absence of this association.^23^,^25^

No association between diabetes and poor tibial run-off was found either. This is in contrast to the presence of association between diabetes and CLI however the role of diabetic microangiopathy in the pathogenesis of trophic lesions should to be taken in account. Conversely, other studies reported a significant association between diabetes and poor run-off.^22^,^24^

Eventual association between diabetes and location of lesions in the SFA was evaluated with a near-significant association (p = 0.051) with proximal SFA involvement. Bakken et al. found an association with mid tract SFA or popliteal involvement in the CLI subgroup.^24^

Regarding to the choice of the device, an interesting association between absence of diabetes and use of DCB was found. We preferred to take advantage of the potential of drug coated balloons in those lesions which would certainly have benefitted from this. In cases of highly calcified lesions or those that didn’t respond to simple PTA, more often, like those of diabetics, we were most likely to use a stent. The use of stents was prevalent among diabetics, even in the absence of significance. The Belgian Diabetic IN.PACT Trial reported excellent results using DCB among diabetics,^33^ although another study considered diabetes as a predictor of late-lumen-loss after DCB in SFA.^34^ Kay et al. found a relationship between Oral Hypoglycemic Agents and patency failure after stenting of SFA but no association between insulin-dependent diabetes and patency.^21^ Another study found diabetes to be an independent factor of patency loss at 6 months after stent positioning in SFA due to long chronic occlusions.^29^ The DEFINITIVE LE Trial reported 12-month patency rates between diabetics and non-diabetics similar after a directional atherectomy in femoral popliteal disease among claudicants.^34^

In the present study almost all the diabetics received a multivessel treatment more frequently on infrapopliteal vessels to improve run-off. A possible explanation could be the major rate of CLI among diabetics which means that a more aggressive approach was managed.

### Limits

The most probable bias of our conclusion is that patients with lesions which were considered complex from an endovascular point of view more often received a “bypass first” approach while an “endovascular first” treatment was chosen for patients with lesions which were considered more responsive, independently of the presence of diabetes itself. Therefore, the diabetic patients in our study had less complex lesions and endovascular procedures most probably had outcomes comparable to the non-diabetics.

Another limit is the relative shortness of the mean follow up time. Unfortunately, several cases of death during the postoperative period were recorded. Many of these patients had already presented clinical conditions at time of hospitalization. On the other hand, the choice of an endovascular procedure in these patients was precisely dictated by their poor conditions.

Measurements of quantity and distribution of calcifications in the SFA lesions using scores such as PACSS were not executed the reason being the unavailability of a preoperative CTA in a fair number of cases. In these latter patients the first preoperative diagnostic exam was the Arteriography.

## V. CONCLUSIONS

In the current study perspective was voluntarily altered and attention was focused on the role of diabetes itself rather than starting from the characteristics of arterial lesions or from clinical conditions of the limb. Our study found a substantial similarity of outcome between diabetics and non-diabetics both in the short and long term. The sole presence of diabetes itself did not significantly influence the results whereas other factors like lesion characteristics seem to have played an important role. This highlights the importance of the appropriate selection of patients to undergo endovascular procedures for femoral popliteal disease. An “endovascular first” approach should be carefully examined after careful investigation of arterial lesions, local conditions of limbs and general status. Our experience tells us that patients who are judged not suitable for endovascular procedure should directly receive, when possible, an open surgical intervention. Proper selection could potentially lead to satisfactory results of endovascular treatment on SFA, even in diabetic patients, who are, by definition, more difficult to treat. Thus, it is highly desirable that new scores be created, to better define which patients could be offered an endovascular procedure on SFA. Moreover, further research is needed to clarify the role of diabetes in this field of Vascular Surgery particularly defining at which level and how could exert its influence on outcome.
